# Antioxidant and longevity inducing properties of coconut water on human dermal fibroblasts

**DOI:** 10.1016/j.heliyon.2024.e41010

**Published:** 2024-12-06

**Authors:** Sarun Na Nakorn, Hasaya Dokduang, Nisana Namwat, Poramate Klanrit, Arporn Wangwiwatsin, Bundit Promraksa, Sirinya Sitthirak, Tinnapat Seaban, Watcharin Loilome

**Affiliations:** a Department of Biochemistry, Faculty of Medicine, Khon Kaen University, Khon Kaen, Thailand; b Department of Systems Biosciences and Computational Medicine, Faculty of Medicine, Khon Kaen University, Khon Kaen, Thailand; c Cholangiocarcinoma Research Institute, Faculty of Medicine, Khon Kaen University, Khon Kaen, Thailand; d Faculty of Medicine, Mahasarakham University, Mahasakham, Thailand; e Regional Medical Sciences Center 2 Phitsanulok, Department of Medical Sciences, Ministry of Public Health, Phitsanulok, Thailand

**Keywords:** Coconut water, Antioxidants, Longevity, Metabolomic

## Abstract

Coconut water is a popular drink in tropical countries and worldwide due to its delicious taste, easy consumption and nutritionally rich properties. Our study aimed to identify bioactive compounds of coconut varieties and their antioxidant as well as longevity effects in 2 different groups of coconuts. These include the bleeding coconut varieties, which are currently most available in the market, namely the Ban Phaeo and Ratchaburi coconut varieties, and the traditional coconut varieties, including Kon-jib and Sampran coconut varieties. Proton nuclear magnetic resonance (^1^H NMR) was used to profile the metabolites in coconut water which revealed 27 metabolites including sugar, organic acids, fatty acids, flavonoids and phenolic compounds. A multivariate data analysis demonstrated an organic acid and phenolic metabolites as the antioxidant bioactive metabolites in coconut water. In addition, the coconut varieties had higher antioxidant bioactive metabolites compared to traditional coconut varieties, while traditional coconut varieties had higher sugar levels. Furthermore, the varieties containing higher antioxidants bioactive metabolites were chosen to examine the longevity effect on human dermal fibroblasts (HDFs). The results revealed that coconut water can significantly decrease cytosolic oxidation in hydrogen peroxide treated HDFs cell line and mediated longevity of fibroblast cells by modulating the expression of proteins in longevity pathway. Data from our study indicate that coconut water serves as source of antioxidants which can be mediated longevity of dermal cell. Moreover, this study provides the useful information for the coconut water production and distribution business.

## Highlights

• Proton nuclear magnetic resonance (^1^H NMR) was used to profile the metabolites in coconut water which revealed 27 metabolites in including sugar, organic acids, fatty acid, flavonoid and phenolic compounds.
• A multivariate data analysis demonstrated an organic acid and phenolic metabolites as the antioxidant bioactive metabolites in coconut water.
• Coconut water reduces cytosolic oxidation and promotes fibroblast longevity by modulating proteins in the longevity pathway.

## Graphical abstract

**Figure:**
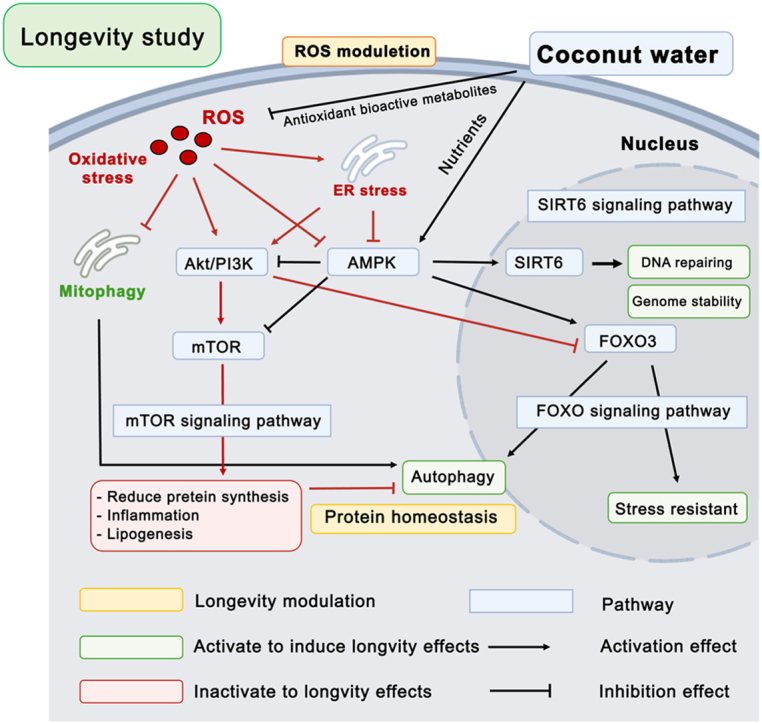

## 1 Introduction

*Cocos nucifera*, or coconut, is an economically significant palm species widely grown in tropical regions. This cross-pollinated plant is not purebred, as each tree results from natural breeding [1]. Coconut have sweeter, aromatic coconut water [2] that contains sugar, minerals, amino acids, enzymes, organic acids, fatty acids, vitamins, and some phenolic compounds. These provide several benefits such as a nutrition source, anti-inflammatory effects, and antioxidant activity. Coconut water from green dwarf coconut varieties has been reported to contain higher levels of antioxidants compared to other coconut varieties [3]. In addition, there are several studies that demonstrated the important health benefits of coconut water to prevent reactive oxygen species (ROS) in fibroblast cell lines by decreasing cytosolic oxidation after exposure to hydrogen peroxide (H_2_O_2_) [4]. Thailand ranks as the third-largest coconut producer globally, renowned for its aromatic coconuts, which are celebrated for their high-quality coconut water. The distinct fragrance and sweet taste of Thai coconut water, along with its nutrient content, including organic acids and phenolic compounds, contribute to its popularity [5]. Different coconut varieties exhibit subtle variations in taste, flavor, and nutrient composition, which are influenced by climate, soil type, growing region, and other environmental factors. These inherent differences are harnessed in biotechnological approaches, where breeding programs focus on hybrid cultivars to improve coconut productivity, resulting in high-yielding varieties with early maturity and larger fruit. Breeding techniques have increased the number of fruits produced by coconut cultivars by more than 45 % [6] than compared with traditional coconut varieties for cultivation. Based on dwarf with dwarf hybrids between coconut varieties were developed to accumulate desirable precocity genes for higher rate of fruits production [7]. However, these factors slightly affected the general composition of coconut water.

Although coconut water is recognized for its nutritional and antioxidant benefits, particularly in green dwarf varieties, there is limited understanding of how different coconut varieties, including those derived from traditional breeding and hybridization, influence metabolite composition and antioxidant properties. The specific impact of these breeding variations on health benefits, particularly in relation to antioxidant activity and longevity, remains underexplored, especially in Thai coconut varieties, despite Thailand's prominence as a major coconut producer.

This study aims to investigate and compare the bioactive metabolites in coconut water from various Thai coconut varieties, including both traditionally bred and hybrid cultivars. Using metabolomic analysis and multivariate data approaches, the research seeks to evaluate the antioxidant activity and potential health benefits of coconut water, focusing on its ability to promote longevity and reduce oxidative stress in human fibroblast cell lines.

## 2 Materials and methods

### 2.1 Chemicals and reagents

Chemicals and reagents used in this study are as follows:2,2-Diphenyl-1-picrylhydrazyl (DPPH), 2,4,6-tripyridyl-s-triazine (TPTZ), sodium acerate, sodium hydroxide (NaOH), hydrochloric acid (HCl), ferric chloride hexahydrate (FeCl_3_6H_2_O), 6-hydroxy-2,5,7,8-tetramethylchroman-2-carboxylic acid (Trolox), gallic acid, sodium deoxycholate, polyvinylidene fluoride membrane and skin milk were purchased from Sigma-Aldrich (St Louis, MO, USA). Folin-Ciocalteau reagent and potassium dihydrogen phosphate (KH_2_PO_4_) (Merck Millipore, Darmstadt, Germany), sodium percarbonate (Na_2_CO_4_) (Carlo Erba, Milan, Italy), acetic acid (AnalaR, Lutterworth, UK), CM-H2DCFDA (2′,7′-Dichlorodihydrofluorescein diacetate or General Oxidative Stress Indicator) and Dulbecco's Modified Eagle's medium (DMEM) were purchased from Thermo Scientific (Rockford, IL, USA), ethanol (RCl labscan, RCI Labscan Limited, Thailand), penicillin-streptomycin, bovine serum albumin (BSA) and trypsin-EDTA form Life technology (Grand Island, NY, USA), Enhanced chemiluminescence plus solution (ECL) was purchased from GE healthcare (Buckinghamshire, UK), deuterium oxide (D_2_O) (ManiSolv, Switzerland) and trimethylsilyl propanoic acid (TSP) was purchased form CIL,USA.

The primary antibodies (ab), including mouse anti-mouse β actin antibody, anti-rabbit mTOR antibody (AB32028), anti-rabbit FOXO3 antibody (ab109629), anti-rabbit SIRT6 antibody (ab191385) were purchased from Abcam (Cambridge, UK), anti-rabbit AMPK antibody (5831S) and anti-rabbit Akt antibody (4685S) form Cell signaling (Massachusetts, USA). The secondary antibodies including anti-mouse antibody and anti-rabbit antibody were purchased from Sigma and Invitrogen, respectively.

### 2.2 Coconut water collection and preparation

The samples of 4 different coconut varieties were collected from Ratchaburi province, Thailand during October 2021. Coconut water in this study was collected from 2 different Thai coconut strains; 1) the bleeding coconut variety, which widely available in the market including the Ban Phaeo coconut variety (assigned as CV1), Ratchaburi coconut variety (assigned as CV2), and the traditional coconut varieties namely, the Kon-jib coconut variety (assigned as CV3) and Sampran coconut variety (assigned as CV4). Five fruits of each coconut variety were collected. Sample characterization was represented (S1). Coconut water samples were prepared by filtered 2 times through Whatman no. 4 filter paper and 0.2 μm filter then stored at −20 °C until used.

### 2.3 Cell lines

A Human dermal fibroblasts (HDFs) cell line was purchased from American Type Culture Collection (ATCC). The cell line was cultured in DMEM nutrient mixture supplemented with 10 % fetal bovine serum and 100 IU/mL of penicillin-streptomycin in a humidified incubator with 5 % CO_2_ atmosphere.

### 2.4 Total phenolic content (TPC) assay

Total phenolic content in coconut water was determined by a Folin–ciocalteu assay. Forty-five μl of coconut water was added to a 96 well plate and 115 μl of 0.05 N folin–ciocalteu reagent was added to the samples, which was incubated for 30 min at room temperature with light protection. After that, 90 μL of 7 % Na_2_CO_4_ was added to stop the reaction. The absorbance of all samples was measured at 765 nm by using a EZ Read 2000 microplate reader (Biochrom, US). Gallic acid was used as the standard compound in this assay. The total phenolic contents in coconut water were calculated as gallic acid equivalent (GAE) from a calibration curve (0–115 μg/mL) and the data were expressed as μg of gallic acid equivalent per 1 mL of sample (μg GAE/mL).

### 2.5 1, 1-Diphenyl-2-picrylhydrazyl (DPPH) free radical scavenging assay

Breifly,100 μL of 400 μM DPPH reagent was added to 100 μL of coconut sample in a 96 well plate, which was then incubated at room temperature with light protection for 30 min. Subsequently, the absorbance was determined at 517 nm. Absolute ethanol was used as a negative control, and 100 μg/ml Trolox in ethanol was used as a positive control. The percentage scavenging radical DPPH activity was calculated [8].

### 2.6 Ferric reducing antioxidant power (FRAP) assay

The FRAP reagent was prepared by mixing 300 mM sodium acetate buffer pH 3.6, 10 mM TPTZ in 40 mM HCl, and 20 mM FeCl_3_•6H_2_O at a ratio of 10:1:1. Aliquots of sample of 100 μL were mixed with 100 μL of FRAP reagent in a 96 well plate. The mixtures were incubated at room temperature with light protection for 5 min. The absorbance of all samples was measured at 593 nm. Trolox was used as standard compound in this assay. Antioxidant activity was reported as microgram of Trolox equivalent per 1 ml of sample (μg TEAC/mL).

### 2.7 Detection of intracellular reaction oxygen species in human dermal fibroblast cell

Human dermal fibroblast cells were plated in black wall 96-well plates 2500 cells in 100 μl of DMEM medium per well for 18 h. Then, these were pre-treated with 100 μl of the designed concentration (0.1 %, 1 % and 10 % v/v) of coconut water in culture media for 48 h. After 48 h, the medium was removed, and cells were washed twice by PBS and replaced with DMEM medium containing 100 mM of H_2_O_2_ with or without the designed concentrations of coconut water and incubated for 5 min. After the incubation time, culture medium was removed, and cells were washed with PBS. To determine intracellular reaction oxygen species,100 μL of medium containing 5 μM of CM-H2DCFDA was added to cell and incubated for 45 min. Subsequently, the medium was removed and 100 μl of PBS was added. The CM-H2DCFDA fluorescence signal was detected by a microplate reader (microplate reader: Tristar 5 Berthold, German).

### 2.8 ^1^H NMR base metabolomic analysis of coconut water

For NMR based metabolomic analysis, the samples of coconut water were mixed with NMR buffer (4.67 mM Na_2_HPO_4_, 0.8 mg/mL TSP, 4 % NaN_3_, 0.2 % v/v D_2_O and adjust pH to 7.4 by HCl and NaOH) at ratio 1:1. The mixture of a sample was then transferred into a 5 mm NMR tube (Duran, Germany). ^1^H NMR spectra were acquired using 400 MHz (Bruker, USA). For data preprocessing of the chemical shift, baseline correction and phasing adjust peak alignment, normalization, and scaling were performed by the R statistical software (Statistical Computing version 3.4.0, Austria) via the metabom8 and ASIC package. Additionally, multivariate statistical analyses were carried out using the SIMCA-P+ version 14.1 (Umetrics Inc., Sweden). The data were mean centered and scaled to Pareto. After preprocessing, the metabolites were identified by the statistical total correlation spectroscopy (STOCSY) method and compared to NMR data base via Chenomx profiler (Chenomx software, Canada) and searched on online metabolite database including, biological magnetic resonance data bank (BMRD) and human metabolite database (HMDB). Moreover, the integration area was identified and used to calculate concentration of the metabolite [9]. A Pearson's correlation and heatmap was performed using MetaboAnalyst 6.0 [10], a free metabolomics data analytical tool available online (https://www.metaboanalyst.ca).

### 2.9 Western blot analysis

Human dermal fibroblast cells were treated with 0.1 %, 1 % and 10 % v/v coconut water for 48 h, cell pellets were harvested and lysed by NP40 lysis buffer (0.1 % SDS, 0.5 % Sodium deoxycholate, 50 mM Tris, 1 % Tween 20) containing protease cocktail inhibitor (Roche, Switzerland) then centrifuged at 12,000 rpm for 10 min to collect protein lysates. The concentration of protein was determined by BCA protein assay kit (Thermo Fisher Scientific, UK). Protein lysates (40 μg) were dissolved in SDS buffer and boiled at 95 °C for 5 min. Protein samples were electrophoresed on 10 % w/v SDS-polyacrylamide gel electrophoresis and transferred to a polyvinylidene fluoride membrane. Non-specific binding on membranes was blocked with 5 % w/v skin milk in Tris-buffer saline (TBS) at room temperature for 1 h, and incubated with primary antibody for Akt, AMPK, mTOR, FOXO3, SIRT6 at 4 °C overnight. After the incubation time, membranes were washed with TBS containing 0.1 % polyoxy ethylene sorbitan monolaurate (Tween-20 or TBS-T) and incubated with horseradish peroxidase-conjugated secondary antibody at room temperature for 1 h. Human β-actin was used as a loading control. The membranes were exposed to the ECL and immunoblot was analyzed by ImageQuantTM Imager (GE Healthcare UK Ltd., UK). The densities of the bands were determined using Image J software version 18.0 (NIH, USA).

### 2.10 Statistical analysis

The antioxidant activity including total phenolic content, percentage scavenging radical DPPH activity, antioxidant assessment, cytosolic oxidation percentage, and longevity effect was presented as a mean ± standard deviation (SD) from three independent experiments. The data were tested with Shapiro-Wilk test for normality's test and analyzed by ANOVA using Fisher's LSD test in GraphPad Prism (GraphPad Software Inc., USA). The multivariate analysis was performed by SIMCA-P+ software. A *p*-value <0.05 was considered as statistically significant. For metabolomic analysis, data bases form STOCSY, BMRD, HMDB and Chenomx were used to confirm and identify the metabolite assignment.

## 3 Results

### 3.1 Phenolic content and antioxidant activity assessments of coconut water

**Fig. 1:**
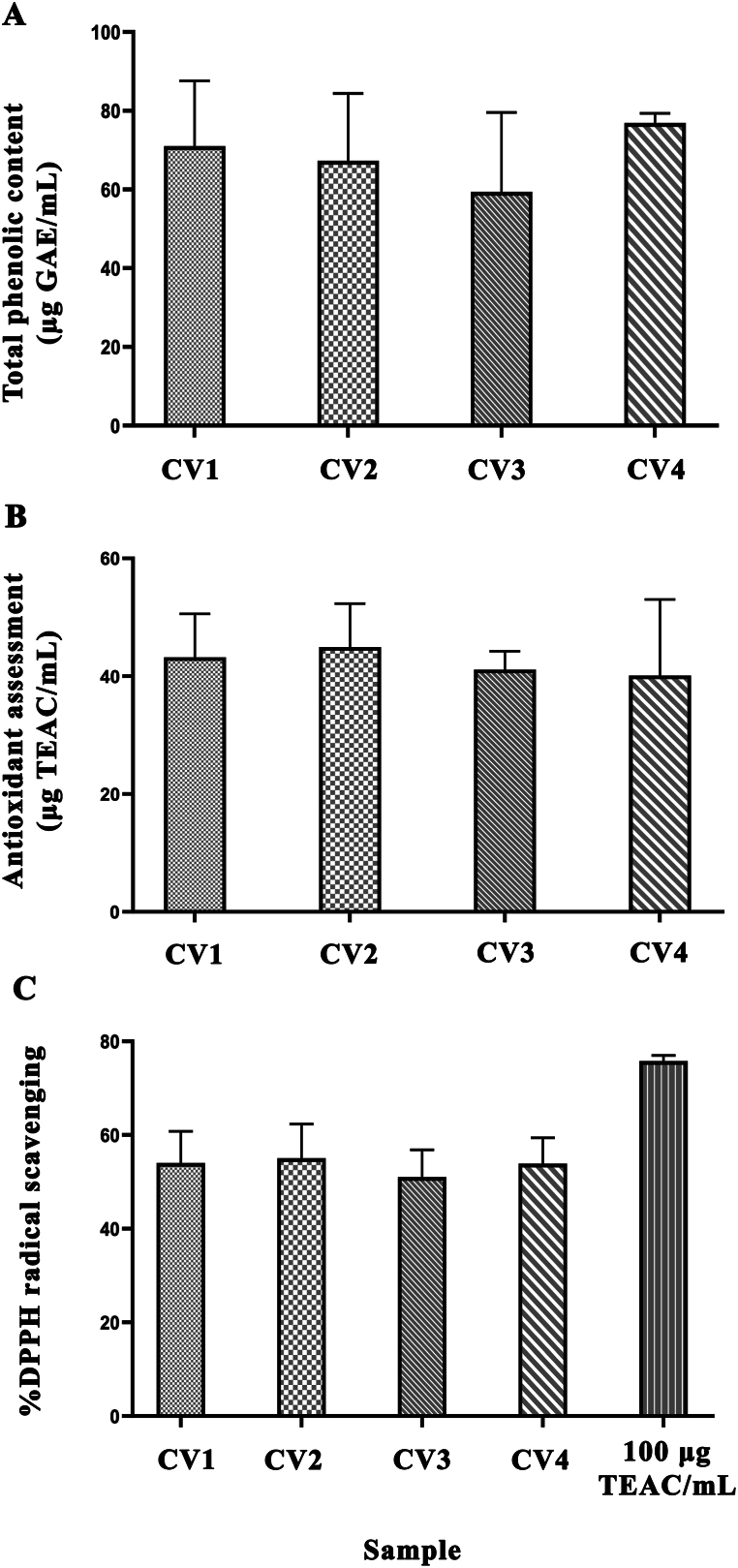
(A) Total phenolic content, (B) Antioxidant assessment of coconut water and (C) DPPH free radical scavenging with Trolox standard from 4 different coconut varieties. Data are represented in means ± SD of three independent experiments (n = 5).

Total phenolic content (TPC) in coconut water was measured by Folin–ciocalteu colorimetric assay. Gallic acid was used as the standard for TPC, as shown in Fig. 1A, the breeding variety coconut water (CV1 and CV2) was 71.03 and 67.30, traditional coconut water (CV3 and CV4) was 59.44 and 76.89 GAE/mL, respectively. Radical scavenging activity of coconut water which was assessed by DPPH assay revealed that all coconut water samples possess a similar antioxidant activity ranging from 51 % to 55 % radical scavenging (Fig. 1B) and FRAP assay (Fig. 1C). Results of all of coconut water samples displaying antioxidant activity are presented no statistically significant differences were found of total phenolic and antioxidant activity of coconut water between the two coconut varieties.

### 3.2 Intracellular reaction oxygen species in human dermal fibroblast cells after treatment with coconut water

**Fig. 2:**
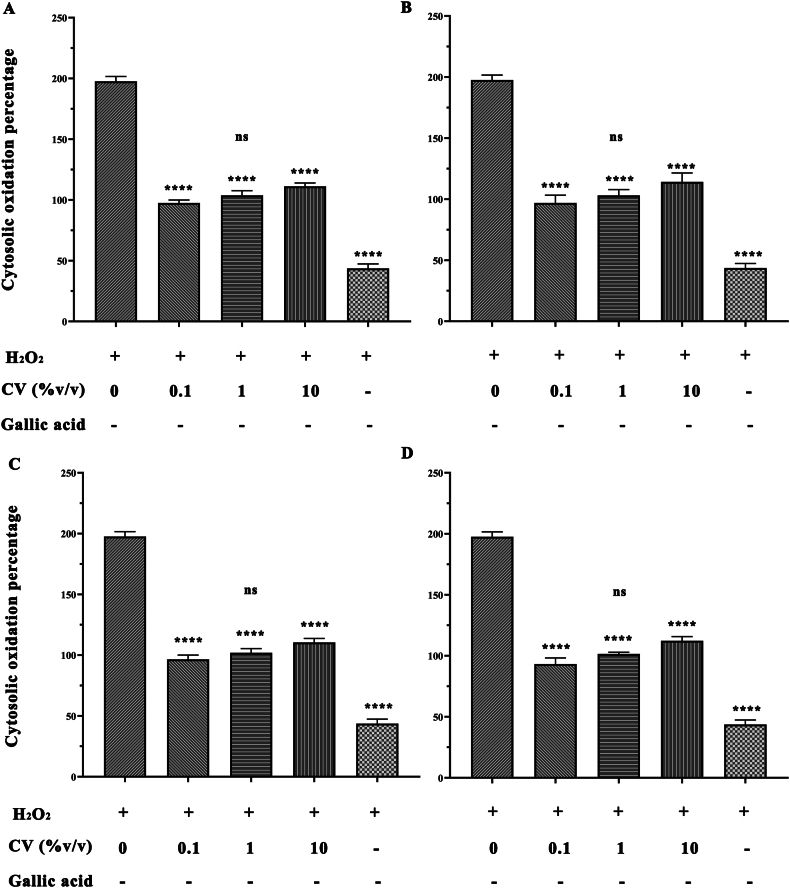
Percentage of cytosolic oxidation in HDFs when combined with H_2_O_2_, coconut water and gallic acid. The HDFs cell were treated with 4 different coconut varieties represented in (A) CV1 (B) CV2 (C) CV3 and CV4 coconut varieties. Data are presented as means ± SD of three independent experiments (n = 5). Different letters (∗) indicate significant differences (*p* < 0.05).

The percentage cytosolic oxidation in human dermal fibroblast cells after being treated with coconut water was measured using the CM-H2DCFDA method, which is used as an indicator for reactive oxygen species (ROS) in cells. The experiments were carried out on 5 fruits of each of the coconut varieties in triplicate and three independent experiments were performed. The results demonstrated coconut water can significantly reduce (*p* < 0.05) cytosolic oxidation caused by H_2_O_2_ in HDFs cells, as shown in Fig. 2A-D. The results showed no statistically significant differences of antioxidant activity of coconut water among different coconut varieties.

### 3.3 NMR data preprocessing and metabolomic clustering of coconut waters

**Fig. 3:**
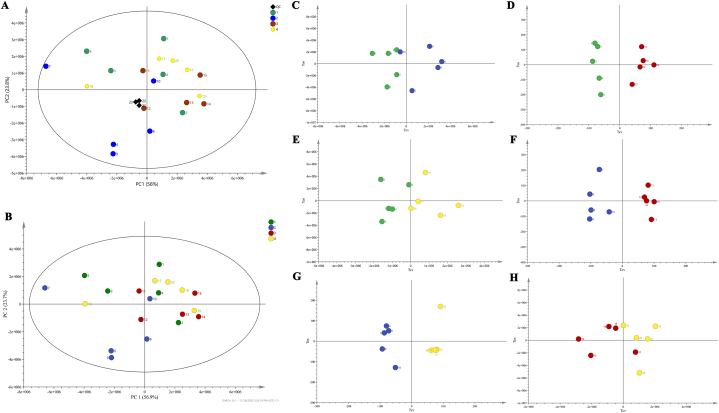
A comparison of the metabolic similarities and variations observed in the 4 varieties of coconut water. PCA score plot of A) all coconut water with QC and B) all coconut water form 4 varieties that represent in green, blue, red, and yellow respectively and O-PLS of coconut water (C) CV1 versus CV2 (D) CV1 versus CV3 (E) CV1 versus CV4 (F) CV2 versus CV3 (G) CV2 versus CV4 and (H) CV3 versus CV4.

The proton nuclear magnetic resonance (^1^H NMR) spectroscopy technique was used to study the metabolites of the four different coconut varieties. The proton nuclear magnetic resonance spectrum, which includes chemical shift and coupling constants, provides quantitative data of intermolecular and intramolecular resonance relationships [9] was obtained. The NMR data preprocessing, including the reference of the chemical shift, baseline correction and phasing adjust peak alignment, normalization, and scaling was performed. Then, the PCA plots, which is an unsupervised model and multivariate data analysis of variables were performed to observe trends, correlation, clusters, and outliers of the samples. Subsequently, PCA plots of coconut water metabolites from different coconut varieties were constructed. As shown in Fig. 3A, a clustering of quality control (QC) samples demonstrated no analytical variation with high analytical precision (R2 = 0.799 Q2 = 0.727). Moreover, the results of the goodness of fit (R2) and goodness prediction (Q2) (Fig. 3B, R2 = 0.805 Q2 = 0.734) confirmed that this clustering model was acceptable for the biological samples [13]. The PCA demonstrated that the coconut water from different coconut varieties can be separated by the first principal component (PC1 = 56.9 %) (Fig. 3B). Six pairwise OPLS-DA models were constructed to compare differences between coconut varieties based on the type of metabolites and their concentration, as shown in Fig. 3C-H.

### 3.4 Metabolomic profiling of coconut waters

**Fig. 4:**
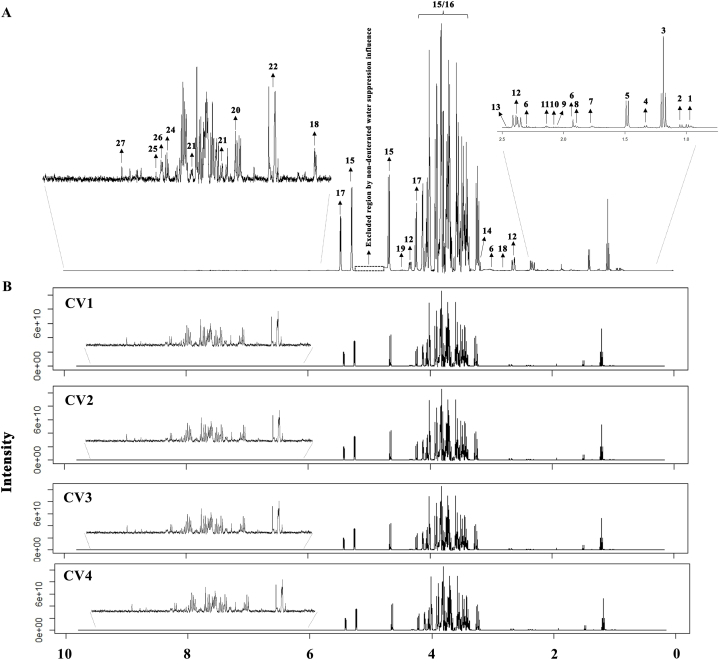
(A) The representative ^1^H NMR spectra of coconut water (Key metabolites: (1) Leucine, (2) Valine Leucine, (3) Ethanol, (4) Lactic acid, (5) Alanine, (6) Gamma-Aminobutyric acid (GABA), (7) Oleic acid, (8) Acetic acid, (9) Peracetic acid, (10) D-ribose, (11) Acetone, (12) Malic acid, (13) Succinic acid, (14) Edetic acid, (15) Glucose, (16) Fructose, (17) Sucrose, (18) Genistein, (19) Formate, (20) Caffeic acid, (21) Gallic acid, (22) Coumarin, (23) Guanine, (24) Oxypurinol, (25) Xanthine, (26) Quercetin, and (27) Formic acid (B) The representative ^1^H NMR spectra of coconut water from 4 different coconut varieties.

The main metabolite compositions of coconut water were investigated using ^1^H-NMR-based metabolic profiling. The metabolite assignment of correlated resonances and statistical total correlation spectroscopy (STOCSY) was employed and searched against online metabolite databases, such as human metabolome database (HMDB), biological magnetic resonance data bank (BMRD) and Chenomx software. The proton resonances at aliphatic region (δ^1^H = 0.5–3.0) including (1) aliphatic organic compounds such as ethanol and acetone, (2) fatty acids compound such as oleic acid, (3) organic acid compounds such as lactic acid, acetic acid, peracetic acid, malic acid, succinic acid, edetic acid, and r-aminobutyric acid (GABA) and (4) some amino acids, including leucine, valine, and alanine were identified respectively. The presence of sugars (δ1H = 2.5–5.0) including D-ribose, glucose, fructose, and sucrose. Moreover, the nitrogen compound, flavonoids and phenolic compounds were detected including genistein, caffeic acid, gallic acid, coumarin, guanine, oxypurinol, xanthine, and quercetin (δ1H = 6.0–8.0), in addition formate and formic acid were identified (δ1H = 4.5 and 8.45). A total 27 identified metabolites are shown in Fig. 4A.

The intensity of ^1^H NMR revealed both qualitative and quantitative variations among the metabolites in different coconut samples. The metabolites in terms of relative abundance and comparison of concentrations are shown in Table 1 and Fig. 4B. Results obtained showed that there were differences of the level of metabolites in organic acids and phenolic compounds, including GABA, acetic acid, formic acid, caffeic acid, gallic acid, and quercetin respectively. These were predominately found in propagated than traditional coconut varieties, while traditional varieties had higher levels of sugar including glucose and fructose and some organic acid namely, peracetic acid, malic acid and succinic acid.

**Table 1:** Quantitative metabolites in coconut water from 4 different coconut varieties.

Metabolites	Category	Metabolites concentration (μM)			
		CV1	CV2	CV3	CV4
Leucine	Amino acid	3.78 ± 0.53	3.31 ± 0.35	3.66 ± 0.43	3.57 ± 0.925
Valine		4.62 ± 0.70	4.01 ± 0.41	4.40 ± 0.49	4.28 ± 1.106
Alanine		58.51 ± 10.03	53.65 ± 4.84	76.91 ± 5.36	60.78 ± 7.325
Ethanol	Aliphatic organic compound	152.62 ± 39.16	141.83 ± 40.53	106.00 ± 6.95	84.04 ± 20.674
Acetone		2.48 ± 1.09	1.96 ± 0.56	1.25 ± 0.22	2.01 ± 0.119
Formate		14.72 ± 2.14	13.43 ± 2.72	15.70 ± 3.53	14.54 ± 1.297
Oleic acid	Fatty acid	0.34 ± 0.13	0.34 ± 0.14	0.26 ± 0.05	0.34 ± 0.027
Lactic acid	Organic acid	5.26 ± 0.76	5.55 ± 0.83	4.92 ± 1.07	4.83 ± 0.632
GABA^a^		14.58 ± 2.52	14.09 ± 6.68	15.70 ± 2.67	10.15 ± 2.800
Acetic acid^a^		3.81 ± 0.60	6.58 ± 4.99	5.45 ± 1.78	2.87 ± 0.553
Peracetic acid^b^		1.21 ± 0.31	0.84 ± 0.17	1.41 ± 0.23	2.04 ± 0.417
Malic acid^b^		18.09 ± 10.62	24.91 ± 4.83	40.79 ± 12.36	25.64 ± 5.659
Succinic acid^b^		1.67 ± 0.64	1.76 ± 0.35	1.70 ± 0.45	2.51 ± 1.153
Edetic acid		21.19 ± 4.44	22.35 ± 4.46	20.51 ± 4.78	26.72 ± 2.817
Formic acid^a^		1.38 ± 0.36	1.23 ± 0.40	1.19 ± 0.24	0.51 ± 0.484
Ribose	Sugar	3.14 ± 0.29	2.90 ± 1.15	3.21 ± 0.31	3.11 ± 0.276
Sucrose^b^		53.19 ± 11.12	54.87 ± 12.54	52.01 ± 14.48	66.43 ± 7.361
Glucose^b^		213.15 ± 49.87	184.14 ± 53.32	239.07 ± 12.20	252.64 ± 13.032
Fructose		162.32 ± 20.93	119.49 ± 66.09	216.73 ± 45.25	214.23 ± 72.158
Genistein	Phenolic	0.03 ± 0.02	0.03 ± 0.02	0.04 ± 0.01	0.04 ± 0.020
Caffeic acid^a^		0.05 ± 0.01	0.06 ± 0.02	0.03 ± 0.02	0.06 ± 0.012
Gallic acid^a^		1.11 ± 0.17	1.0 ± 0.42	0.78 ± 0.23	0.83 ± 0.25
Quercetin^a^		0.17 ± 0.02	0.14 ± 0.05	0.16 ± 0.05	0.13 ± 0.025
Coumarin	Flavonoids	2.13 ± 2.36	2.00 ± 0.77	0.96 ± 0.28	1.53 ± 0.369
Guanine	Nitrogenous compound	0.34 ± 0.13	0.14 ± 0.09	0.20 ± 0.05	0.29 ± 0.171
Oxypurinol		0.54 ± 0.38	0.53 ± 0.15	0.41 ± 0.06	0.34 ± 0.195
Xanthine		0.08 ± 0.04	0.09 ± 0.05	0.17 ± 0.20	0.12 ± 0.136

a Metabolites presented in breeding than traditional coconut varieties.

b Metabolites presented in traditional than breeding coconut varieties, data are represented in means ± SD (n = 5).

### 3.5 Identification of antioxidant activity-related bioactive metabolites in coconut water

**Fig. 5:**
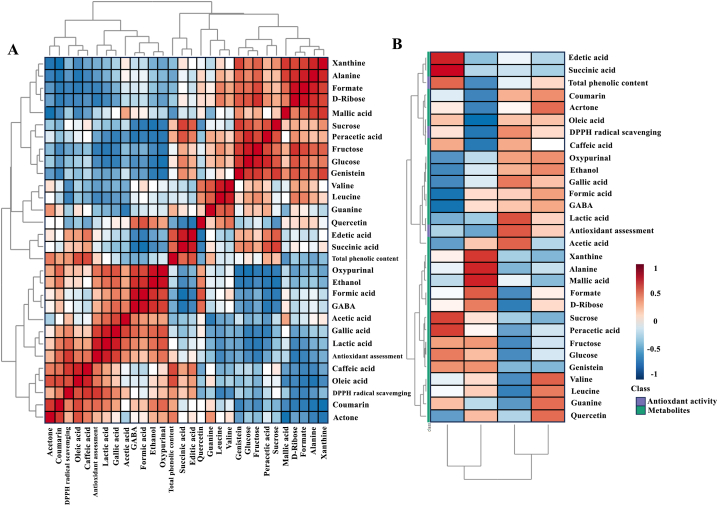
Correlation analysis of metabolites and antioxidant of coconut water A) The overall Pearson's correlation between various coconut water metabolites and antioxidant bioactivities. Positive correlations are indicated in shades of red, whereas negative correlations are indicated in shades of blue. B). Heatmaps were and obtained between various coconut varieties between the concentration of metabolites and antioxidant bioactivities from coconut water. Positive (red) or negative (blue) values indicate higher or lower concentrations of the metabolites in either group of comparison.

To determine the antioxidant activity-related bioactive metabolites in coconut water an overall Pearson's correlation was preformed to evaluate the linear relationship between pairs of variables. Fig. 5A shows the positive correlation between metabolites with total phenolic content, DPPH radical scavenging activity and antioxidant assessment. Pearson's correlation showed positive correlations between the studied bioactivities and identified metabolites. These results demonstrate the relationship of these phenolics with the antioxidant activity (DPPH and FRAP) of coconut water. Antioxidant -bioactive metabolites also had a high correlation of phenolic and organic acid metabolites in coconut water. The heatmap result (Fig. 5B) supported that the CV1 and CV2, which are breeding coconut varieties, had high values of antioxidant-bioactive metabolites, while CV3 and CV4 which are traditional coconut varieties had high levels of sugar and organic metabolites.

### 3.6 Longevity inducing effect of coconut water on human dermal fibroblast cell

**Fig. 6:**
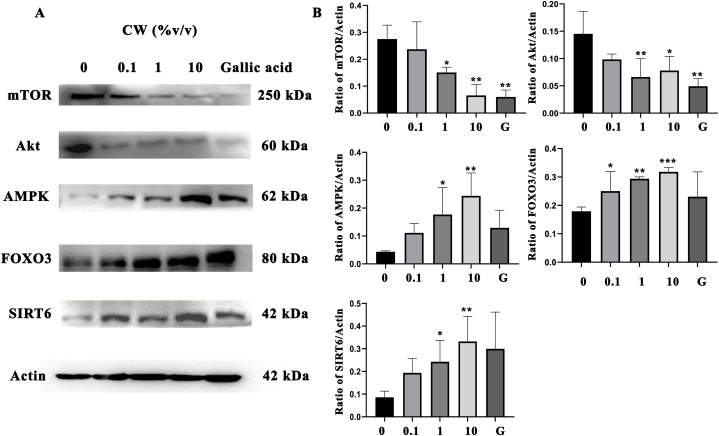
Effect of coconut water on HDFs cells that are related to expression of protein A) The Western blot analysis revealed AMPK, Akt, mTOR, SIRT6, and FOXO3 protein expression B) The band signal intensity ratio of AMPK, Akt, mTOR, SIRT6, and FOXO3 protein per actin. Data are represented in means ± SD of three independent experiments.

Metabolomic results showed that coconut water from propagated varieties have high levels of antioxidant activity and antioxidant-bioactive metabolites. As such, the coconut water from breeding varieties were treated on HDFs cells to investigate the effect of coconut water on the expression of longevity related protein expression including, AMPK, Akt, mTOR, SIRT6, and FOXO3, respectively. As shown in Fig. 6A, coconut water significantly decreased the expression of mTOR and Akt proteins while increasing the expression of AMPK protein (Fig. 6B). These changes subsequently led to a significant increase in FOXO3 expression, a key component of the longevity pathway involving autophagy. Moreover, the expression of SIRT6 protein involved in longevity via DNA repairing pathway was found to be increased in HDFs cells treated with coconut water when compared with gallic acid as the positive control.

## 4 Discussion

Our study identified metabolites and biological activities related to the antioxidant and longevity effects of coconut water from four Thai coconut varieties. These varieties were classified into two groups: commercially available types (Ban Phaeo, CV1, and Ratchaburi, CV2) and traditional types (Kon-jib, CV3, and Sampran, CV4). All varieties contained high levels of total phenolics, with no statistically significant differences in phenolic content. Antioxidant activity was assessed using two methods: the DPPH radical scavenging assay (electron transfer-based) and the FRAP assay (hydrogen atom transfer-based). The results demonstrated high activity of coconut water in both the FRAP and DPPH radical scavenging activity assay due to its ability to donate an electron. There was no statistically significant difference of antioxidant activity of coconut water from different coconut varieties.

Generally, ROS can be produced during the activity of amphibolic metabolic pathways in the inner mitochondrial membrane and can result in the creation of free radicals [11] which play an upstream role to induce oxidative stress on cells. Therefore, the inhibitory effect of reactive oxygen species (ROS) production of coconut water on reducing human dermal fibroblast cells (HDFs) due to H_2_O_2_-treatment was further investigated for oxidative stress to cells [12]. Our results showed that coconut water can significantly reduce the level of cytosolic oxidation percentage in HDFs cells induced by hydrogen peroxide. However, no statistically significant differences of antioxidant activity on HDFs cells of coconut water from different coconut varieties was found. Notably, the high concentration of coconut water (%v/v) shown the reduction of activity to protection of coconut water against ROS production. A previous study has demonstrated that coconut water from the green dwarf variety contains long chain fatty acids, such as oleic and palmitic acids, therefore there is a possibility of β-oxidation occurring at the peroxisome level, resulting in a slight rise in peroxide [4].

Metabolomics and chemometric approaches were used in our study to detect subtle changes in a large dataset with comprehensive metabolite measurements [13]. An unsupervised PCA showed that coconut water from different coconut varieties can be separated by principal component (PC1) analysis. Individual comparisons using supervised. OPLS-DA revealed that coconut water from breeding varieties (CV1 and CV2) could be clearly separated from traditional varieties (CV3 and CV4). Furthermore, a total 27 metabolites were identified in all coconut water, which included amino acid, sugar, flavonoid, aliphatic organic, nitrogenous. In addition new organic acids including edetic acid, succinic acid, paracetic acid and phenolic metabolites including, genistein, caffeic acid, gallic acid, and quercetin were also detected than have been detected in previous studies ([14,15]). The quantitative variations among the metabolite were performed through quantitative NMR (qNMR) [16]. The results demonstrated that traditional coconut varieties contained higher concentrations of sugar while breeding coconut varieties had higher concentrations of amino acid and phenolic metabolites. Moreover, multivariate data analysis showed that 11 metabolites were related to antioxidant-bioactive metabolites. These results are consistent with previous studies that have revealed the significance of antioxidant activity of these metabolites namely acetone, ethanol, caffeic acid [17], gallic acid [18], guanine [19], coumarin, alanine [20], acetic acid [21], oxypurinol [22], lactic acid [23], oleic acid [24], and formic acid [25], respectively. In addition, ethanol and acetone proved to be the best solvents to extract compounds with antioxidant capacity [26]. The present study demonstrated that breeding coconut varieties had higher antioxidant-bioactive metabolites than traditional coconut varieties.

**Fig. 7:**
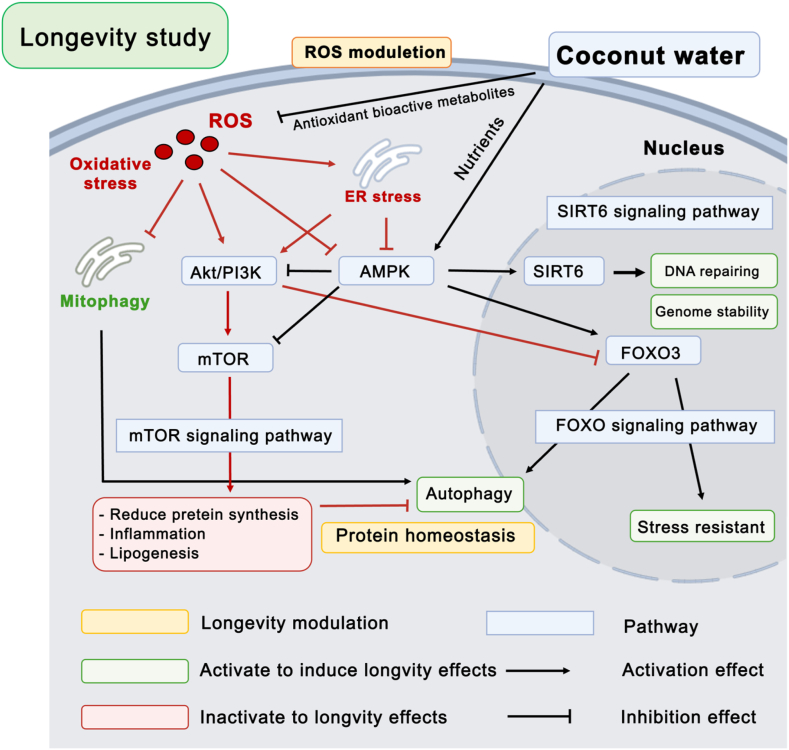
Summary of longevity effects of coconut water on Human dermal fibroblasts.

The results from antioxidant activity tests and the metabolomic study demonstrated coconut water contains essential nutritional compounds that have antioxidant bioactive metabolites that can prevent ROS production on HDFs cells. The ROS are byproducts of normal cellular function that are generated in a number of cellular compartments. An over production of ROS has an effect on unhealthy aging [27] and longevity because oxidative stress can lead to functional and morphological impairment in cells including ER stress and mitophagy [28]. The excessive accumulation of ROS can lead to activation of the mammalian target of rapamycin (mTOR) signaling pathway though protein kinase B (Akt) [29]. The activation of mTOR plays roles in cellular energy status and hypoxia stress, extracellular signals which include growth factors and amino acids [30] resulting in decreased anabolic processes, such as translation, ribosomal biogenesis, protein synthesis rate, and decreased catabolic processes, such as autophagy, which promote cellular growth and proliferation [31]. The inhibition of mTOR through the AMPK (5′ AMP-activated protein kinase) mechanism acts as a sensor of cellular energy that responds to nutrition. It functions to restore energy homeostasis by activating catabolic pathways and inhibiting cell division and development [32] leading to the inhibition of many pathological conditions such as inflammation, diabetes, aging, and cancer [34]. AMPK can also regulate the expression of mTOR and in turn reduce expression of Akt protein [33]. AMPK and upregulated levels of total and nuclear Forkhead box O (FOXO) [34] also play roles in longevity pathways including, induce autophagy and stress resistant, which impact the age and lifespan of an organism [35]. Moreover, AMPK is also highly associated with the sirtuin family that upregulate the levels the expression of SIRT6, an accelerated aging disorder that leads to senescence, maintains the structure of telomere chromatin, prevents genomic instability after DNA damage, and protects cells from senescence [36]. The results of this study showed that coconut water has reduced expression of mTOR and Akt protein and increased expression of AMPK, FOXO3 and SIRT6 which play roles in longevity activity to induce autophagy, stress resistant, DNA repairing to induce genome stability. In summary of longevity effects of coconut water on HDFs cell were shown on Fig. 7.

While this study identified bioactive metabolites in coconut water from various Thai coconut varieties, along with its antioxidant activity and potential to promote longevity and reduce oxidative stress in human fibroblast cell lines, the longevity induction properties have been demonstrated using and *in vitro* model. In order to confirm the role of coconut water in longevity induction, other models including *in vivo* models should be further examined to validate the underlying molecular mechanisms.

## 5 Conclusions

Coconut water has antioxidant properties and biological effects in protecting against oxidative damage on human dermal fibroblasts after exposure to oxidative stress. Results from our metabolomic study showed that coconut water from the breeding varieties had higher antioxidant-bioactive metabolites and lower sugar levels compared to coconut water from the traditional varieties. Therefore, coconut water currently available in the market provides a healthy drink in terms of antioxidant and longevity induction.

## CRediT authorship contribution statement

**Sarun Na Nakorn:** Writing – original draft, Visualization, Validation, Methodology, Investigation, Formal analysis, Data curation. **Hasaya Dokduang:** Writing – review & editing, Supervision, Methodology. **Nisana Namwat:** Writing – review & editing, Supervision, Methodology, Investigation. **Poramate Klanrit:** Writing – review & editing, Supervision, Methodology. **Arporn Wangwiwatsin:** Writing – review & editing, Supervision, Methodology. **Bundit Promraksa:** Writing – review & editing, Methodology. **Sirinya Sitthirak:** Writing – review & editing, Methodology. **Tinnapat Seaban:** Writing – review & editing, Methodology. **Watcharin Loilome:** Writing – review & editing, Supervision, Project administration, Methodology, Investigation, Funding acquisition, Formal analysis, Data curation, Conceptualization.

## Ethics approval and consent to participate

This work (HE671273) be exempt, the Khon Kaen University Ethics Committee for Human Research (KKUEC)

## Availability of data and materials

The data of coconut information, total phenolic and antioxidant activity, ROS detection in HDFs cell of each coconut sample and PPM form STOCSY were shown in supplementary data. The Metabolomic data can be accessed at Open Science Framework (OSF): https://osf.io/j64eu/.

## Funding Statement

This work was supported by the NSRF under the Basic Research Fund of 10.13039/501100004071Khon Kaen University and Invitation Research Grant, 10.13039/501100010804Faculty of Medicine, 10.13039/501100004071Khon Kaen University (IN63331) to WL.

## Declaration of competing interest

The authors declare that they have no known competing financial interests or personal relationships that could have appeared to influence the work reported in this paper.

## References

[1] Yong J.W.H., Ge L., Ng Y.F., Tan S.N. (2009). The chemical composition and biological properties of coconut (cocos nucifera L.) water. Molecules.

[2] Dasanayaka P.N., Everard J.M.D.T., Karunanayaka E.H., Nandadasa H.G. (2009). Analysis of coconut (Cocos nucifera L.) diversity using microsatellite markers with emphasis on management and utilisation of genetic resources. J Natl Sci Found.

[3] Zhang B. (Oct. 2019). Effects of organic acids, amino acids and phenolic compounds on antioxidant characteristic of zhenjiang aromatic vinegar. Molecules.

[4] Santos J.L.A. (2013). Evaluation of chemical constituents and antioxidant activity of coconut water (Cocus nucifera L.) and caffeic acid in cell culture. An. Acad. Bras. Cienc..

[5] Luckanatinvong V., Mahatheeranont S., Siriphanich J. (Mar. 2018). Variation in the aromatic nature of Nam-Hom coconut depends on the presence and contents of 2-acetyl-1-pyrroline. Sci. Hortic..

[6] Yousefi K., Abdullah S.N.A., Hatta M.A.M., Ling K.L. (May 2023). Genomics and transcriptomics reveal genetic contribution to population diversity and specific traits in coconut. Plants.

[7] Arhin L., Abdullah S. nor A., Jaafar J.N., Izan Ramlee S. (Sep. 2023). Conventional and modern breeding technologies for improving dwarf coconut cultivars: a review. J. Hortic. Sci. Biotechnol..

[8] Baliyan S. (Feb. 2022). Determination of antioxidants by DPPH radical scavenging activity and quantitative phytochemical analysis of Ficus religiosa. Molecules.

[9] Bharti S.K., Roy R. (May 2012). Quantitative 1H NMR spectroscopy. TrAC, Trends Anal. Chem..

[10] Pang Z. (Apr. 2024). MetaboAnalyst 6.0: towards a unified platform for metabolomics data processing, analysis and interpretation. Nucleic Acids Res..

[11] Newsholme P., Cruzat V.F., Keane K.N., Carlessi R., De Bittencourt P.I.H. (2016). Molecular mechanisms of ROS production and oxidative stress in diabetes. Biochem. J..

[12] Shi X. (2021). Exploring the protective and reparative mechanisms of G. Lucidum polysaccharides against H2O2-induced oxidative stress in human skin fibroblasts. Clin Cosmet Investig Dermatol.

[13] Steuer A.E., Brockbals L., Kraemer T. (2019). Metabolomic strategies in biomarker research-new approach for indirect identification of drug consumption and sample manipulation in clinical and forensic toxicology?. Front. Chem..

[14] Porto E. (May 2020). Ozone and plasma processing effect on green coconut water. Food Res. Int..

[15] Sucupira N.R., Alves Filho E.G., Silva L.M.A., de Brito E.S., Wurlitzer N.J., Sousa P.H.M. (Feb. 2017). NMR spectroscopy and chemometrics to evaluate different processing of coconut water. Food Chem..

[16] Zhang Y.Y. (Jun. 2021). Quantitative 1H nuclear magnetic resonance method for assessing the purity of dipotassium glycyrrhizinate. Molecules.

[17] Purushothaman A., Babu S.S., Naroth S., Janardanan D. (2022). Antioxidant activity of caffeic acid: thermodynamic and kinetic aspects on the oxidative degradation pathway. Free Radic. Res..

[18] Daglia M., Lorenzo A., Nabavi S., Talas Z., Nabavi S. (Aug. 2014). Polyphenols: well beyond the antioxidant capacity: gallic acid and related compounds as neuroprotective agents: you are what you eat. Curr Pharm Biotechnol.

[19] Gudkov S.V., Shtarkman I.N., Smirnova V.S., Chernikov A.V., Bruskov V.I. (May 2006). Guanosine and inosine display antioxidant activity, protect DNA in vitro from oxidative damage induced by reactive oxygen species, and serve as radioprotectors in mice. Radiat. Res..

[20] Zúñiga-Núñez D. (Jan. 2018). Atypical antioxidant activity of non-phenolic amino-coumarins. RSC Adv..

[21] Neffe-Skocińska K., Karbowiak M., Kruk M., Kołożyn-Krajewska D., Zielińska D. (Aug. 2023). Polyphenol and antioxidant properties of food obtained by the activity of acetic acid bacteria (AAB) – a systematic review. J. Funct.Foods.

[22] Augustin A.J., Boker T., Blumenroder H., Lutz J., Spitznas M. (Oct. 2024). Free radical scavenging and antioxidant activity of allopurinol and oxypurinol in experimental lens-induced uveitis. Invest. Ophthalmol. Vis. Sci..

[23] Hu Y. (May 2023). Lactic acid bacteria with a strong antioxidant function isolated from “Jiangshui,” pickles, and feces. Front. Microbiol..

[24] Wei C.C., Yen P.L., Chang S.T., Cheng P.L., Lo Y.C., Liao V.H.C. (Jun. 2016). Antioxidative activities of both oleic acid and camellia tenuifolia seed oil are regulated by the transcription factor DAF-16/FOXO in Caenorhabditis elegans. PLoS One.

[25] Treichel J.L., Henry M.M., Skumatz C.M.B., Eells J.T., Burke J.M. (Nov. 2004). Antioxidants and ocular cell type differences in cytoprotection from formic acid toxicity in vitro. Toxicol. Sci..

[26] Borges A., José H., Homem V., Simões M. (Feb. 2020). Comparison of techniques and solvents on the antimicrobial and antioxidant potential of extracts from Acacia dealbata and olea europaea. Antibiotics.

[27] Snezhkina A.V. (2019). ROS generation and antioxidant defense systems in normal and malignant cells. Oxid. Med. Cell. Longev..

[28] Su L., Zhang J., Gomez H., Kellum J.A., Peng Z. (2023). Mitochondria ROS and mitophagy in acute kidney injury. Autophagy.

[29] Lawrence J., Nho R. (2018). The role of the mammalian target of rapamycin (mTOR) in pulmonary fibrosis. Int. J. Mol. Sci..

[30] Wang Y., Fung N.S.K., Lam W.C., Lo A.C.Y. (Jul. 2022). mTOR signalling pathway: a potential therapeutic target for ocular neurodegenerative diseases. Antioxidants.

[31] Pan H., Finkel T. (Apr. 2017). Key proteins and pathways that regulate lifespan. J. Biol. Chem..

[32] Vara-Ciruelos D., Russell F.M., Grahame Hardie D. (Jul. 2019). The strange case of AMPK and cancer: dr jekyll or mr hyde? †. Open Biol.

[33] Garza-Lombó C., Schroder A., Reyes-Reyes E.M., Franco R. (Apr. 2018). mTOR/AMPK signaling in the brain: cell metabolism, proteostasis and survival. Curr Opin Toxicol.

[34] Dai B. (Oct. 2017). ASIC1a promotes acid-induced autophagy in rat articular chondrocytes through the AMPK/FoxO3a pathway. Int. J. Mol. Sci..

[35] Sun X., Chen W.D., Wang Y.D. (Aug. 2017). DAF-16/FOXO transcription factor in aging and longevity. Front. Pharmacol..

[36] Li X. (Mar. 2021). SIRT6 in senescence and aging-related cardiovascular diseases. Front. Cell Dev. Biol..

