# Supplementary material for: Antioxidant and longevity inducing properties of coconut water on human dermal fibroblasts
Source: Heliyon. 2024 Dec 6;10(24):e41010. doi: 10.1016/j.heliyon.2024.e41010 (PMC11696670; doi:10.1016/j.heliyon.2024.e41010)
Supplement: Multimedia component 1 [file mmc1.docx]

# Supplements data

## Supplementary data 1. Detail information of coconut used in this study.

**Table S1** Information on coconut samples from different coconut varieties used in this study.

| Coconut  varieties | Sample | Weight of coconut fruit (kg) | Coconut water volume (mL) | Age  (week) | Harvesting date |
| --- | --- | --- | --- | --- | --- |
| Ban Phaeo | 1 | 1.57 | 200 | ~4 | 03/09/2022 |
|  | 2 | 2.2 | 400 |  |  |
|  | 3 | 3.4 | 440 |  |  |
|  | 4 | 2.264 | 300 |  |  |
|  | 5 | 2.06 | 350 |  |  |
| Ratchaburi | 1 | 2.225 | 330 |  |  |
|  | 2 | 2.629 | 340 |  |  |
|  | 3 | 2.555 | 150 |  |  |
|  | 4 | 2.6 | 270 |  |  |
|  | 5 | 2.553 | 220 |  |  |
| Kon-Jib | 1 | 1.935 | 280 |  |  |
|  | 2 | 1.823 | 280 |  |  |
|  | 3 | 1.535 | 200 |  |  |
|  | 4 | 2.345 | 200 |  |  |
|  | 5 | 1.879 | 320 |  |  |
| Sampran | 1 | 2.546 | 400 | ~3.5 | 04/09/2022 |
|  | 2 | 2.456 | 390 |  |  |
|  | 3 | 2.333 | 220 |  |  |
|  | 4 | 2.34 | 375 |  |  |
|  | 5 | 2.596 | 400 |  |  |

## Supplementary data 2. Phenolic content and antioxidant activity of coconut water

**Table S2** Total phenolic content and antioxidant activity of coconut water from 4 different coconut varieties

| Coconut varieties | Sample | Total phenolic content (ug/mL of gallic acid) | % DPPH radical scavenging (%SA) | Antioxidant assessment | |
| --- | --- | --- | --- | --- | --- |
|  |  |  |  | Galic acid (µg/mL) | Trolox  (µg/mL) |
| Ban Phaeo | 1 | 60.90±1.14 | 42.78±2.53 | 6.117±0.17 | 37.564±4.44 |
|  | 2 | 66.61±4.26 | 56.73±5.67 | 7.673±0.75 | 47.186±5.36 |
|  | 3 | 52.74±4.93 | 52.80±2.64 | 6.788±0.67 | 41.686±4.05 |
|  | 4 | 80.51±11.54 | 57.75±5.28 | 8.749±0.41 | 53.805±3.37 |
|  | 5 | 94.41±1.86 | 59.96±5.97 | 5.840±0.17 | 35.839±2.07 |
|  | Average | 71.03 16.53 | 54.00±6.79 | 7.034±1.19 | 43.216±7.36 |
| Ratchaburi | 1 | 50.12±7.93 | 59.62±5.94 | 7.108±0.35 | 43.669±2.74 |
|  | 2 | 91.42±2.20 | 60.41±6.47 | 8.998±1.11 | 55.441±8.72 |
|  | 3 | 55.73±13.64 | 42.80±2.59 | 6.266±0.62 | 38.447±3.32 |
|  | 4 | 78.16±8.00 | 54.00±5.04 | 6.215±0.50 | 38.096±1.70 |
|  | 5 | 61.05±2.93 | 58.37±6.95 | 7.706±1.24 | 49.096±6.73 |
|  | Average | 67.30±17.09 | 55.04±7.28 | 7.313±1.19 | 44.950±7.38 |
| Kon-Jib | 1 | 87.07±4.28 | 50.40±6.53 | 6.445±0.52 | 39.600±4.13 |
|  | 2 | 39.98±3.50 | 52.23±3.79 | 7.211±0.88 | 44.206±4.36 |
|  | 3 | 47.83±5.51 | 54.48±6.43 | 6.061±0.19 | 37.169±1.06 |
|  | 4 | 48.14±8.55 | 41.59±0.54 | 7.234±0.42 | 44.465±3.68 |
|  | 5 | 74.10±9.03 | 56.49±5.84 | 6.535±0.10 | 40.120±1.53 |
|  | Average | 59.44±20.14 | 51.04±5.76 | 6.697±0.51 | 41.112±3.15 |
| Sampran | 1 | 89.18±5.35 | 54.50±4.75 | 7.872±0.47 | 48.388±3.43 |
|  | 2 | 90.89±4.88 | 51.98±12.08 | 5.561±0.59 | 34.135±4.12 |
|  | 3 | 55.34±4.01 | 45.52±3.75 | 3.309±0.04 | 20.197±1.00 |
|  | 4 | 79.63±10.40 | 58.12±6.86 | 8.284±0.63 | 50.851±2.68 |
|  | 5 | 69.42±6.05 | 59.26±7.54 | 7.764±0.69 | 47.091±2.76 |
|  | Average | 76.89±2.50 | 53.88±5.50 | 6.540±2.09 | 40.133±12.89 |

Data are represented in means ± SD (n=3) from 3 independent experiments

## Supplementary data 3. Percentage of cellular ROS in HDFs cell.

**Table S3** Cytosolic oxidation percentage of human dermal fibroblasts (HDFs) after treated with coconut water form 4 different varieties.

| Sample | No | Cytosolic oxidation percentage | | |
| --- | --- | --- | --- | --- |
|  |  | 0.1% of CW | 1% of CW | 10% of CW |
| Ban Phaeo | 1 | 91.69±5.54 | 100.94±6.58 | 107.48±8.83 |
|  | 2 | 92.56±0.16 | 100.04±4.31 | 109.15±1.98 |
|  | 3 | 96.08±2.91 | 101.82±1.57 | 114.81±8.03 |
|  | 4 | 96.43±2.28 | 101.21±2.13 | 113.7±1.69 |
|  | 5 | 107.71±0.00 | 111.59±2.00 | 125.89±3.06 |
|  | Average | 97.97±3.85 | 102.91±3.38 | 111.86±3.92 |
| Ratchaburi | 1 | 97.332±4.83 | 102.088±5.30 | 106.86±3.15 |
|  | 2 | 93.81±2.61 | 102.283±3.25 | 107.243±1.37 |
|  | 3 | 94.061±3.66 | 101.518±4.44 | 107.292±2.07 |
|  | 4 | 98.017±3.17 | 102.418±2.37 | 109.568±0.51 |
|  | 5 | 99.42±1.46 | 101.428±0.46 | 106.064±2.38 |
|  | Average | 98.02±7.77 | 104.08±8.59 | 114.37±6.44 |
| Kon-Jib | 1 | 91.69±5.54 | 100.94±6.58 | 107.48±8.83 |
|  | 2 | 92.56±0.16 | 100.04±4.31 | 109.15±1.98 |
|  | 3 | 96.08±2.91 | 101.82±1.57 | 114.81±11.03 |
|  | 4 | 96.43±2.28 | 101.21±2.13 | 113.7±1.69 |
|  | 5 | 107.71±0.00 | 111.59±2.00 | 125.89±3.06 |
|  | Average | 101.31±11.88 | 105.83±11.62 | 112.6±9.98 |
| Sampran | 1 | 84.90±3.65 | 100.85±5.48 | 109.20±4.11 |
|  | 2 | 96.05±3.25 | 101.40±4.21 | 109.01±0.13 |
|  | 3 | 93.45±3.09 | 101.47±0.84 | 116.99±4.48 |
|  | 4 | 93.49±1.50 | 103.71±4.38 | 112.2±8.50 |
|  | 5 | 98.03±3.52 | 100.34±4.80 | 114.09±3.97 |
|  | Average | 99.38±9.08 | 105.39±9.84 | 112.22±8.39 |
| 10 µg/mL of Gallic acid | | 42.87±5.88 | | |
| HDFs treat H_2_O_2_ | | 201.05±8.26 | | |

Data are represented in means ± SD (n=3) from 3 independent experiments

## Supplementary data 4. The identification of metabolites detected in coconut water.

**Table S4** ^1^H-NMR signal of identified metabolites in coconut water.

| **Metabolites** | **1H-NMR signal (δ)** |
| --- | --- |
| Alanine | 1.47 and 3.53 |
| Leucine | 0.94, 0.96, 0.98, 1.03, 1.02, 2.04 and 3.56 |
| Valine | 0.94, 1.00, 1.01, 2.35, and 3.68 |
| Ethanol | 1.15 and 3.65 |
| Lactic acid | 1.34 and 4.22 |
| GABA | 1.87, 2.28, 3.00, and 3.01 |
| Acetic acid | 1.90 |
| Oleic acid | 0.94, 1.29, 1.73, 2.28, and 5.36 |
| Peracetic acid | 2.11 |
| D-Ribose | 2.24 |
| Acetone | 2.23 |
| Malic acid | 2.35, 2.69, and 4.29 |
| Succinic acid | 2.39 |
| Edetic acid | 3.25, 3.43, 3.55, and 3.74 |
| Glucose | 3.23, 3.38, 3.43, 3.55, 3.58, 3.67, 3.71, 3.83, 3.89, 4.01, 4.11, 4.21, 5.23 |
| Fructose | 3.58, 3.67, 3.71, 3.98, 4.01 and 4.11 |
| Sucrose | 3.47, 3.54, 3.77, 3.92, 4.04, 4.21 and 5.41 |
| Formate | 4.48 |
| Genistein | 6.39, 6.43, 6.95, 7.23 and 7.62 |
| Caffeic acid | 6.29, 6.93, 7.04 and 7.59 |
| Gallic acid | 6.94, and 7.30 |
| Xanthine | 7.75 |
| Coumarin | 6.40, 7.28, 7.33, 7.47, 7.65 and 7.69 |
| Guanine | 7.60 |
| Oxypurinal | 7.72 |
| Quercetin | 6.435, 6.524, 6.929, 7.129 and 7.657 |
| Formic acid | 8.46 |

## Supplementary data 5. Longevity protein expression (non-adjusted images).


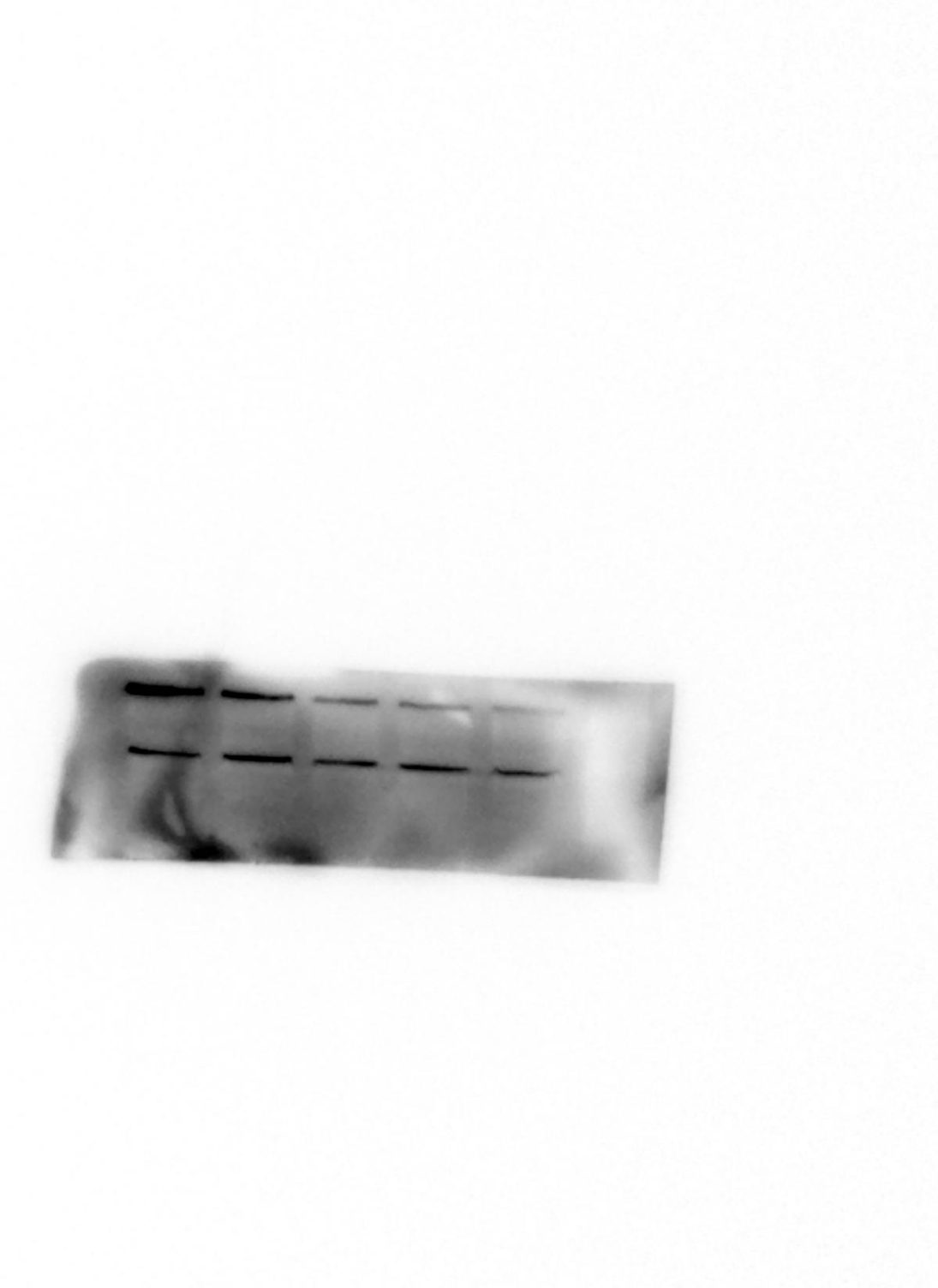


250 KDa

mTOR

Figure 5.1) mTOR protein expression (250 KDa)


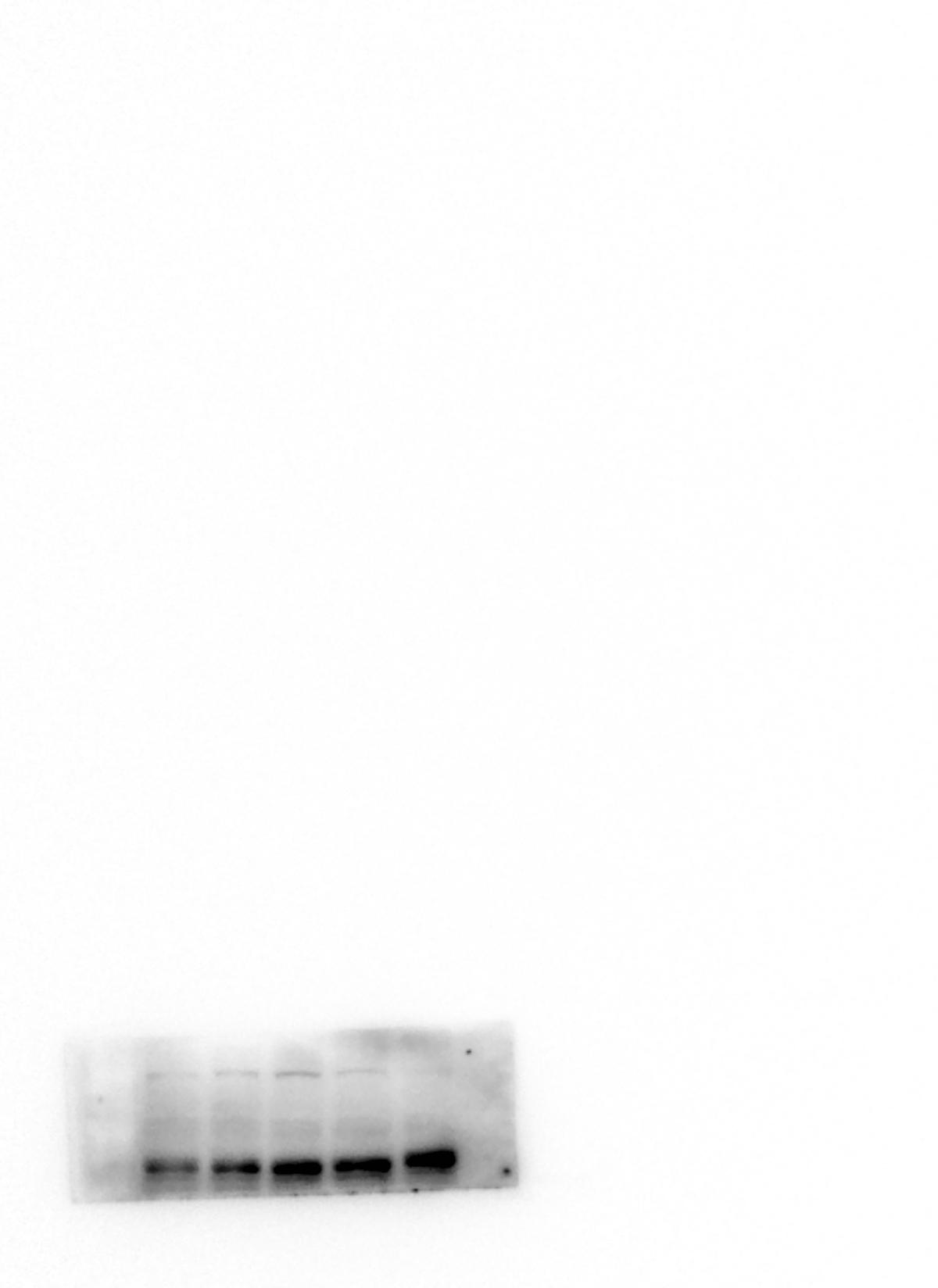


80 KDa

FOXO3

Figure 5.2) FOXO3 protein expression (80 KDa)


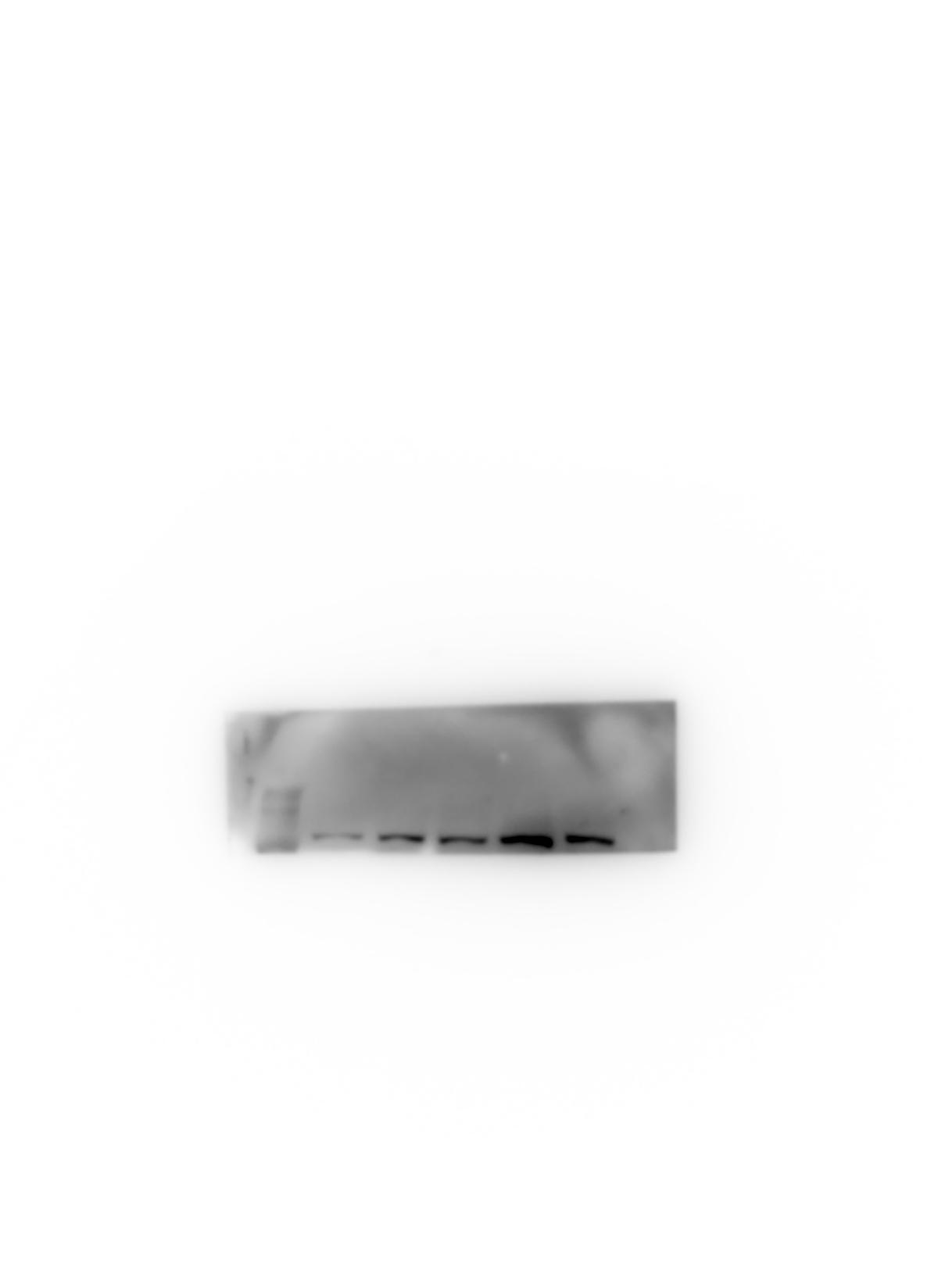


62 KDa

AMPK

Figure 5.3) AMPK protein expression (62 KDa)

## Supplementary data 4. Longevity protein expression (non-adjusted images) (Cont.).


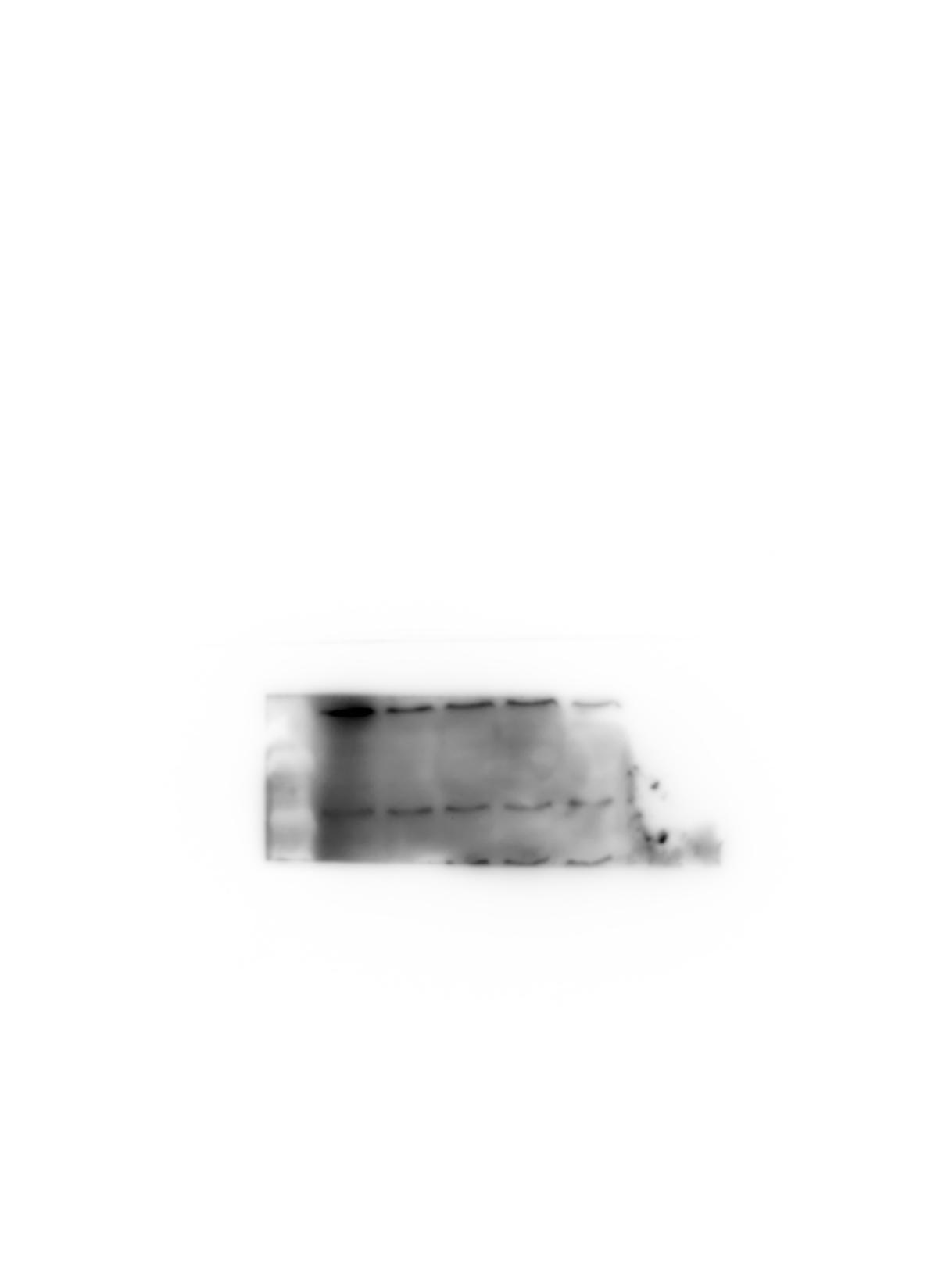


Akt

60 KDa

45 KDa

Figure 5.4) mTOR protein expression (60 KDa)


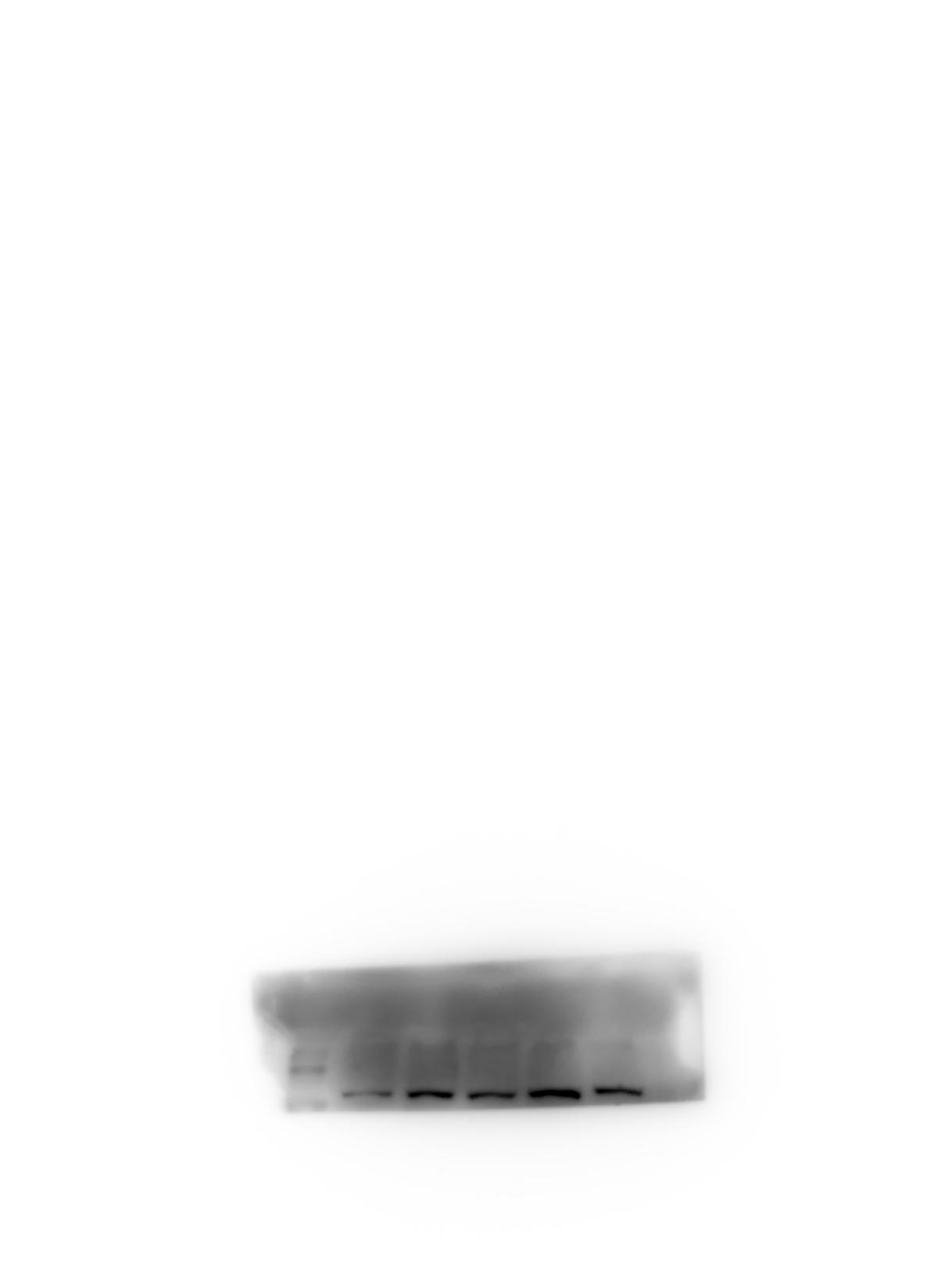


SIRT6

42 KDa

Figure 5.5) SIRT6 protein expression (42 KDa)


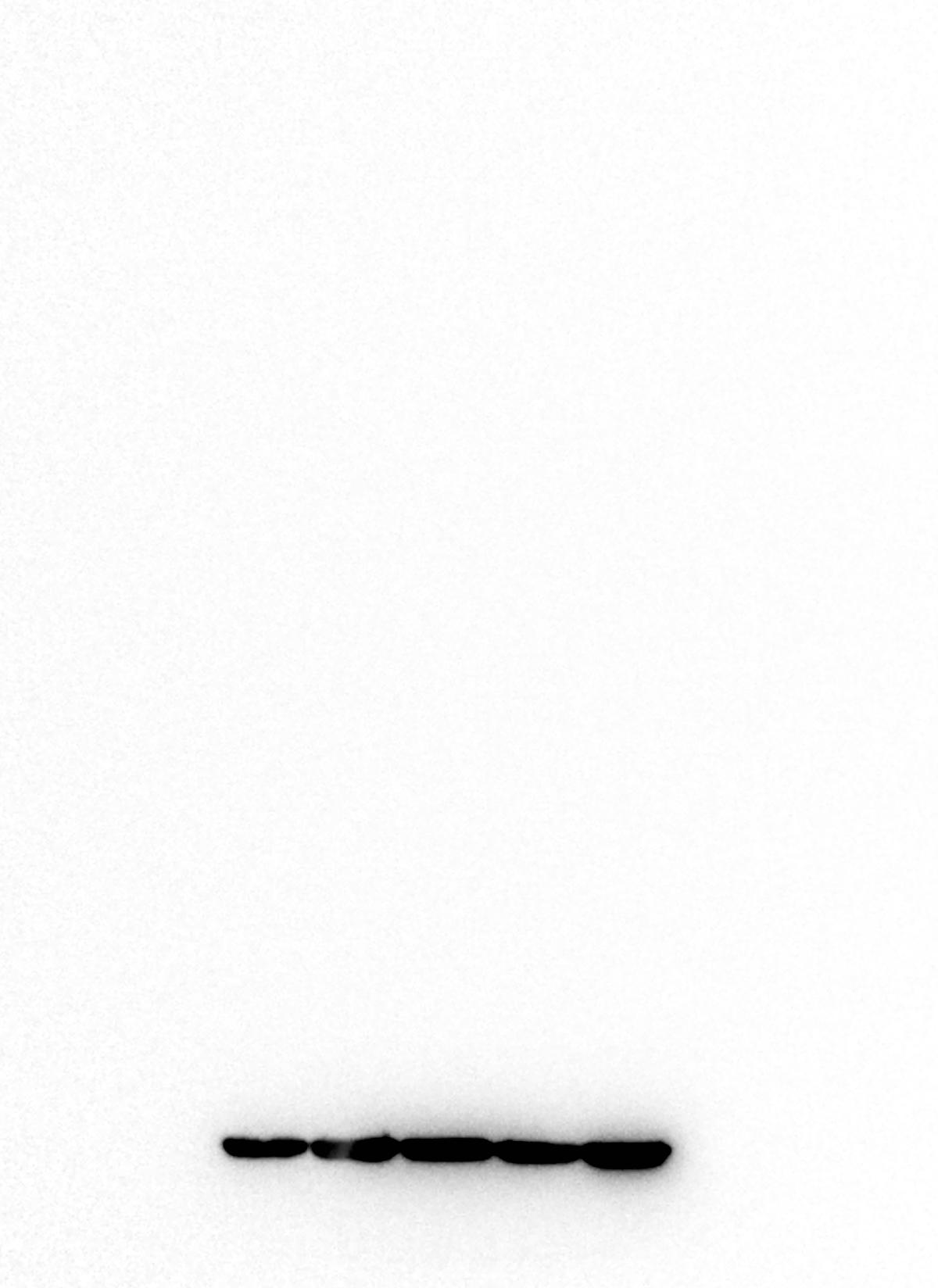


Actin

45 KDa

Figure 5.6) actin protein expression (45 KDa)
